# Comparison of methods of Weaning from Nasal Continuous Positive Airway Pressure in Preterm Infants: A Randomized Controlled Trial

**DOI:** 10.12669/pjms.42.5.15003

**Published:** 2026-05

**Authors:** Rabia Asif, Heena Rais, Tayyaba Anwar, Saba Safdar

**Affiliations:** 1 Rabia Asif, MBBS, Department of Pediatric Medicine, Ziauddin University, Karachi, Pakistan; 2 Heena Rais, FCPS, Department of Pediatric Medicine, Ziauddin University, Karachi, Pakistan; 3 Tayyaba Anwar, FCPS, Department of Pediatric Medicine, Ziauddin University, Karachi, Pakistan; 4 Saba Safdar, DCH, Department of Pediatric Medicine, Ziauddin University, Karachi, Pakistan

**Keywords:** Gestational age, Low birth weight, Preterm, Nasal continuous positive airway pressure, Weaning

## Abstract

**Objective:**

To compare the efficacy of different nasal continuous positive airway pressure (NCPAP) weaning strategies in preterm infants presenting with respiratory distress syndrome (RDS).

**Methodology:**

This randomized controlled trial was conducted at the Department of Pediatrics, Dr. Ziauddin University Hospital, Karachi, Pakistan, from July to December 2024. It included preterm infants with gestational ages between 28 to < 37 weeks and low birth weight (<2500 grams) presenting with RDS. Group-A underwent sudden weaning of NCPAP, Group-B had gradual weaning with time off NCPAP, and Group-C underwent pressure weaning. Outcome measures included weaning success, total days on NCPAP and length of hospital stay. Data were analyzed using IBM-SPSS Statistics, version 26.0.

**Results:**

Seventy-five infants participated, with 39 (52.0%) females, and a mean age of 3.35±3.49 hours. NCPAP weaning was successful in 69 (92.0%) cases. There was no significant difference in weaning success rates among different strategies (p=0.581). The day of NCPAP weaning initiation showed a significant correlation with weaning strategies (p<0.001). The successful weaning day was also significantly correlated with weaning strategies (p=0.001). Duration of NCPAP was significantly different between groups (p=0.002). Hospitalization duration was significantly shorter in the sudden weaning group (8.52±5.65 days) compared to the gradual (12.32±5.73 days) and pressure weaning groups (11.80±5.55 days) (p=0.041).

**Conclusion:**

Infants subjected to sudden NCPAP weaning had a significantly shorter overall duration of NCPAP, leading to a significantly reduced total duration of hospitalization compared to those undergoing gradual or pressure weaning. Success rates for sudden, gradual, and pressure NCPAP weaning varied but without significant differences.

***Trial Registration:*** NCT07009366 (https://clinicaltrials.gov/search?cond=NCT07009366).

## INTRODUCTION

Neonatal mortality is attributed to 46% of under-5 deaths among children worldwide.^1^ Globally, out of a total of 2.7 million neonates who die each year, the majority of them are attributable to complications of prematurity (36%), intrapartum-related hypoxia (25.8%), and sepsis (18.6%).^1^,^2^ Prematurity also influences serious complications like respiratory distress syndrome (RDS), necrotizing enterocolitis (NEC), intraventricular hemorrhage (IVH), and infections 3, and these newborns are more vulnerable to respiration issues following birth.^4^ RDS is the most common cause of respiratory issues among preterm newborns, playing a fundamental role in the morbidity and mortality in these neonates.^5^,^6^ These issues need to be handled with improved management, preventive techniques, and supportive care.^7^

Currently, much attention is being given to non-invasive forms of resuscitation in the delivery room (DR) for premature neonates. Initiation of nasal continuous positive airway pressure (NCPAP) as a non-invasive ventilation method, immediately after birth, is becoming the gold standard approach according to the latest guidelines.^8^,^9^ International Liaison Committee on Resuscitation (ILCOR) 2020 guidelines^10^ also proposed the utilization of NCPAP in lieu of ventilation for the management of RDS in preterm neonates who need respiratory aid after birth in the DR.^4^ Possible complications affiliated with the use of NCPAP include nasal injury, obstruction of airways with mucus, increased risk of pneumothorax,^11^ probable interrelation with IVH, and most commonly gaseous abdominal distention, also called “CPAP belly syndrome”,^12^,^13^ which was at first elucidated in 1992 by Jaile et al.^14^

Optimal weaning from NCPAP is of considerable clinical significance, as inappropriate, repeated, or early weaning trials may ultimately impose lung injury, increased work of breathing, atelectasis, bradycardia, desaturation, and chronic lung injury,^11^,^15^ and hence extended time of ventilation and more use of resources.^6^ There is a need for planned strategies for withdrawal from NCPAP. Some of the weaning strategies have been delineated, which include sudden discontinuation of NCPAP, gradual curtailment by slowly increasing the time off, gradual reduction of pressure, or coalescence of all the above-mentioned methods.^11^,^15^ A study carried out by Rastogi et al.^16^ described sudden weaning as more successful than gradual weaning. Jensen et al described that the number of days on NCPAP, the number of tries it took to successfully stop using NCPAP, or the duration of hospital stay did not vary for sudden vs gradual pressure weaning of NCPAP pressures in newborns.^11^

Pakistan, as well as other developing countries, lacks data regarding the weaning protocol of NCPAP. Ascertaining the best weaning method that could facilitate having a shorter duration on NCPAP and the shortest length of hospital stay is very much needed. It was hypothesized that there is a difference in outcomes among neonates with different weaning strategies of non-invasive ventilation. This study aimed to determine the efficacy of different NCPAP weaning strategies in pre-term infants presenting with respiratory distress and ultimately establish the best method to withdraw from NCPAP.

## METHODOLOGY

This single-blinded, randomized controlled trial was conducted at the Department of Pediatrics, Dr. Ziauddin University Hospital, Karachi, from July to December 2024.

### Ethical Approval:

It was approved by the hospital Ethics Committee with Ref. No. 4880222RAPED, Dated; June 25, 2022).

A sample size of 68 was calculated using OpenEpi based on Rastogi et al.,^16^ with 95% confidence and 80% power, and inflated to 75 (25 per arm). Eligible participants were preterm, low-birth-weight infants (28 to <37 weeks) presenting with respiratory distress confirmed by radiology and the Downes score. Exclusion criteria included major congenital anomalies (e.g., diaphragmatic hernia, CNS malformations, congenital heart disease), intrapartum hypoxia, grade III–IV intraventricular hemorrhage, or low APGAR (<4–5 at five minutes). Major congenital anomalies were excluded on the basis of initial clinical examination and relevant investigations where indicated. Congenital heart disease was ruled out by clinical assessment, with echocardiography performed in suspected cases. Written informed consent was obtained from parents or guardians, and demographic, perinatal, and postnatal data were recorded.

Infants were randomized into three groups as Group-A (sudden weaning), in which NCPAP was withdrawn entirely once stability criteria were met, Group-B (gradual time-off), in which off periods were progressively increased from 1-6 hours twice daily before complete withdrawal, and Group C (pressure weaning), in which CPAP pressure was reduced stepwise by 1 cm H_2_O every 12–24 hours to 5 cm H_2_O before removal. Participants were randomized using a computer-generated random allocation sequence. Group assignment was concealed using sealed opaque envelopes opened at the time of enrolment. Failure of weaning was defined by abnormal ABGs (pH <7.25, PaO_2_ <50 mmHg, PaCO_2_ >60 mmHg), SpO_2_ <87% despite FiO_2_ >60%, recurrent apneas or bradycardia requiring positive pressure ventilation, or severe respiratory distress. Infants who failed were restarted on NCPAP for 24 hours before re-attempting, or shifted to mechanical ventilation if deterioration continued.

All neonates were closely monitored with clinical assessments, pulse oximetry, ABGs, and chest X-ray. Orogastric tubes were used to prevent gastric distension, and supportive therapy including surfactant (INSURE technique), antibiotics, and caffeine was given as indicated. All infants were managed according to a standardized NICU NCPAP care protocol, with uniform use of CPAP equipment/interface, humidification measures, and routine nursing care across the three groups. The primary outcome was successful weaning, while secondary outcomes included duration of NCPAP, hospital stay, and complications. ABGs evaluations were performed when clinically indicated and were interpreted alongside clinical signs and oxygenation status. Weaning failure was diagnosed on the basis of predefined clinical and physiological criteria, with ABG parameters considered when available. Weaning was considered successful when FiO_2_ was 21% with SpO^2^ >90%, CPAP pressure was 5 cm H_2_O, and the infant had stable breathing without apneas requiring intervention. Successful weaning was sustained tolerance after discontinuation of NCPAP without meeting any predefined weaning failure criteria during the subsequent hospital course. The primary comparative analysis was performed on a per-protocol basis. Infants who underwent the assigned weaning strategy and had assessable weaning outcomes were included in the analysis. Infants who died before initiation of weaning were excluded from weaning outcome analysis, as the allocated intervention could not be applied. No crossover occurred between groups. The outcome assessor was blinded to group allocation, while the treating clinical team could not be blinded because of the nature of the interventions. Complications were assessed throughout NICU stay by routine clinical monitoring and relevant investigations where indicated. Nasal trauma was identified on clinical examination, while pneumothorax was confirmed radiologically in suspected cases. Reintubation or escalation to NIPPV/mechanical ventilation was performed based on worsening respiratory distress, recurrent apnea requiring intervention, persistent hypoxemia despite support, or respiratory acidosis on ABGs analysis. Statistical analysis was performed using SPSS version 26. Quantitative variables were reported as mean±SD and categorical variables as frequencies and percentages. One-way ANOVA compared hospital stay and NCPAP duration between groups, and chi-square test assessed associations with complications. A p-value <0.05 was considered significant.

## RESULTS

In a total of 75 babies, 39 (52.0%) were females while 36 (48.0%) were males, representing a female-to-male ratio of 1.1:1. The mean age at the time of presentation was 3.35±3.49 hours, ranging from 1 to 30 hours. There were 33 (44.0%) infants who had an age ≤ 2 hours at the time of presentation, while 42 (56.0%) were aged above two hours. The residential status of 74 (98.7%) infants was urban. The place of admission among 49 (65.3%) infants was the operation room, while the labour room and emergency room were the places of admission among 7 (9.3%) and 19 (25.3%) infants, respectively. The mean maternal age was 28.51±4.47 years, ranging from 20 to 40 years. The mean gestational age was 32.05±2.47 weeks, ranging between 28 and less than 37 weeks. Tables-I and II show a comparison of demographic, clinical, physiological, laboratory and imaging parameters with different NCPAP weaning strategies.

**Table-I:** Comparison of demographic and clinical characteristics of infants with NCPAP weaning strategies (n=75).

Variables		Total	Weaning Strategy			P-value
			Sudden weaning of NCPAP (n=25)	Gradual Weaning with increasing the time off CPAP (n=25)	Pressure weaning of NCPAP (n=25)	
Gender	Male	36 (48.0%)	11 (44.0%)	15 (60.0%)	10 (40.0%)	0.326
	Female	39 (52.0%)	14 (56.0%)	10 (40.0%)	15 (60.0%)	
Age (hours)	≤2	39 (52.0%)	9 (36.0%)	10 (40.0%)	14 (56.0%)	0.321
	>2	36 (48.0%)	16 (64.0%)	15 (60.0%)	11 (44.0%)
Birth weight (kg)		1.81±0.50	1.82±0.52	1.92±0.44	1.66±0.51	0.161
Maternal age (years)		28.51±4.47	28.52±4.74	29.00±4.64	28.00±4.14	0.737
Gestational age (weeks)		32.05±2.47	32.04±2.42	32.36±2.29	31.76±2.73	0.696
Admission place	Emergency room	19 (25.3%)	6 (24.0%)	6 (24.0%)	7 (28.0%)	0.267
	Labor room	7 (9.3%)	1 (4.0%)	5 (20.0%)	1 (4.0%)	
	Operation theatre	49 (65.3%)	18 (72.0%)	14 (56.0%)	17 (68.0%)	
Antenatal history	Pregnancy-induced hypertension	18 (24.0%)	5 (20.0%)	8 (32.0%)	5 (20.0%)	0.518
	Gestational diabetes	14 (18.7%)	4 (16.0%)	6 (24.0%)	4 (16.0%)	0.704
	Intrauterine growth retardation	3 (4.0%)	2 (8.0%)	1 (4.0%)	-	0.353
	Pre-rupture of membranes	31 (41.3%)	40 (40.0%)	12 (48.0%)	9 (36.0%)	0.681
	Fever or antibiotic use	28 (37.3%)	10 (40.0%)	9 (36.0%)	9 (36.0%)	0.945
	Prenatal steroids given	59 (78.7%)	19 (76.0%)	19 (76.0%)	21 (84.0%)	0.728
Mode of delivery	Caesarean section	59 (78.7%)	19 (76.0%)	19 (76.0%)	21 (84.0%)	0.728
	Vaginal delivery	16 (21.3%)	6 (24.0%)	6 (24.0%)	4 (16.0%)	
Post-natal characteristics	Immediate cry	57 (76.0%)	19 (76.0%)	18 (72.0%)	20 (80.0%)	0.803
	Resuscitation done	14 (18.7%)	6 (24.0%)	6 (24.0%)	2 (8.0%)	0.245
	Surfactant given	43 (57.3%)	14 (56.0%)	12 (48.0%)	17 (68.0%)	0.355
	Caffeine given	27 (36.0%)	8 (32.0%)	11 (44.0%)	8 (32.0%)	0.594
	Mechanical ventilation	7 (28.0%)	1 (4.0%)	2 (8.0%)	4 (16.0%)	0.332
Diagnosis among preterm infants	RDS	56 (74.7%)	18 (72.0%)	20 (80.0%)	18 (72.0%)	0.771
	TTN	2 (2.7%)	1 (4.0%)	-	1 (4.0%)	
	Congenital pneumonia	13 (17.3%)	4 (16.0%)	5 (20.0%)	4 (16.0%)	
	Sepsis	4 (5.3%)	2 (8.0%)	-	2 (8.0%)	

**Table-II:** Comparison of clinical and laboratory parameters with NCPAP weaning strategies (n=75).

Variables	Total	Weaning Strategy			P-value
		Sudden weaning of NCPAP (n=25)	Gradual Weaning with increasing the time off CPAP (n=25)	Pressure weaning of NCPAP (n=25)	
Heart rate (min) Range: 99-180	146.59±16.89	143.23±14.78	141.88±17.39	154.64±16.01	0.014
Respiratory rate (min) Range: 33-86	61.56±8.62	62.08±8.05	60.56±9.72	62.04±8.25	0.781
Systolic blood pressure Range: 44-94	64.01±11.60	59.32±10.43	71.44±9.98	61.28±10.87	<0.001
Diastolic blood pressure Range: 22-57	35.61±8.26	33.16±8.24	38.44±8.16	35.24±7.84	0.073
SpO_2_ Range: 86-98	95.03±2.33	95.52±1.58	94.04±3.36	95.52±1.19	0.032
APGAR score	Minute-1	6.52±0.82	6.64±1.0	6.88±0.60	0.309
	Minute-5	7.56±0.58	7.80±0.76	7.80±0.65	0.347
Ballard score	31.99±2.35	31.92±2.34	32.12±2.44	31.92±2.34	0.943
Haemoglobin (g/dl)	15.45±2.64	16.10±1.76	14.30±3.43	15.95±2.13	0.026
TLC	10.18±4.66	10.62±4.59	9.39±4.16	10.54±2.26	0.583
Platelets	237.35±75.80	229.24±67.97	256.64±75.10	226.16±74.12	0.268
CRP	7.80±16.08	4.89±6.36	5.91±11.71	12.51±24.05	0.193
Procalcitonin	6.27±11.22	5.21±12.19	7.34±10.13	6.25±11.61	0.803
ABG pH	7.30±0.11	7.30±0.11	7.29±0.12	7.32±0.11	0.624
PO_2_	157.12±31.54	160.46±25.14	159.42±35.58	151.47±33.45	0.551
PCO_2_	31.61±7.78	31.41±8.26	32.67±7.40	30.76±7.86	0.683
HCO_3_	15.82±4.26	14.90±4.14	15.57±4.51	16.99±4.0	0.208
SpO_2_ Range 92-99.7	96.53±2.36	96.79±2.08	96.96±2.39	95.84±2.53	0.198
Chest Findings	Clear	19 (76.0%)	20 (80.0%)	14 (56.0%)	0.242
	Crepitation	6 (24.0%)	5 (20.0%)	11 (34.0%)	
Ultrasound brain findings	Normal	24 (96.0%)	23 (92.0%)	23 (92.0%)	0.673
	IVH Grade-1	-	1 (4.0%)	1 (4.0%)	
	IVH Grade-2	1 (4.0%)	1 (4.0%)	1 (4.0%)	
Blood culture findings	Positive	3 (12.0%)	6 (24.0%)	4 (16.0%)	0.521
	Negative	22 (88.0%)	19 (76.0%)	21 (84.0%)	

In 72 (96.0%) patients, NCPAP was initiated on day one, while in 3 (4.0%) cases, it was started on day two. The weaning started in 32 (45.7%) patients on the 2^nd^ day, 32 (45.7%) on the 3^rd^ day, while 1 (1.4%) case had initiation of weaning on the 1^st^ day. Overall, successful weaning was noted in 69 (92.0%) patients. Five (6.7%) patients died before the weaning could be started. Reintubation was required in 2 (2.7%) patients. On day one, in all patients (25 in each group), NCPAP was initiated except for 3 (12.0%) patients from the pressure weaning group (p=0.044). Duration of NCPAP was significantly shorter among the sudden weaning group (p=0.002). Table-III shows a comparison of initiation, duration and tapering of NCPAP with respect to different weaning strategies.

**Table-III:** Comparison of initiation, duration, and tapering of NCPAP and weaning success for different weaning strategies of NCPAP (N=75).

Variables		Weaning strategy			P-value
		Sudden weaning of NCPAP (n=25)	Gradual weaning with increasing the time off NCPAP (n=25)	Pressure weaning of NCPAP (n=25)	
Initiation of NCPAP	Day-1	25 (100%)	25 (100%)	22 (88.0%)	0.044
	Day-2	-	-	3 (12.0%)	
Duration of NCPAP (days)	1 day	3 (12.5%)	-	-	0.002
	2 days	13 (54.2%)	2 (9.1%)	7 (29.2%)	
	3 days	7 (29.2%)	12 (54.5%)	12 (50.0%)	
	4 days	1 (4.2%)	8 (36.4%)	5 (20.8%)	
Initiation of NCPAP weaning*	Day-1	-	-	1 (4.2%)	<0.001
	Day-2	21 (87.5%)	3 (13.6%)	8 (33.3%)	
	Day-3	3 (12.5%)	17 (77.3%)	12 (50.0%)	
	Day-4	-	2 (9.1%)	3 (12.5%)	
Weaning successful**	Day-2	16 (66.7%)	2 (9.1%)	6 (26.1%)	0.001
	Day-3	7 (29.2%)	12 (54.5%)	10 (43.5%)	
	Day-4	1 (4.2%)	8 (36.4%)	7 (30.4%)	

* Weaning could not be initiated in 5 patients due to mortality.

** Out of the remaining 6 patients, reintubation was required in 1 patient; 5 patients had already died.

The duration of hospital stay was noted as significantly less with the sudden weaning of the NCPAP strategy than the other two groups (p=0.041). In terms of complications and mortality, all three strategies had statistically similar outcomes (Table-IV).

**Table-IV:** Comparison of duration of hospital stay, complications and mortality for different weaning strategies of NCPAP (N=75).

Study variables		Weaning Strategy			P-value
		Sudden weaning of NCPAP (n=25)	Gradual Weaning with increasing the time off CPAP (n=25)	Pressure weaning of NCPAP (n=25)	
*Duration of hospital stay (days)* *Means ± SD*		8.52±5.65	12.32±5.73	11.80±5.55	0.041
Complications	Intraventricular haemorrhage	1 (4.0%)	3 (12.0%)	3 (12.0%)	0.532
	Necrotizing enterocolitis	1 (4.0%)	4 (16.0%)	2 (8.0%)	0.332
	Pneumothorax	2 (8.0%)	1 (4.0%)	1 (4.0%)	0.768
	Pulmonary haemorrhage	2 (8.0%)	1 (4.0%)	2 (8.0%)	0.807
Mortality	Yes	1 (4.0%)	3 (12.0%)	1 (4.0%)	0.424
	No	24 (96.0%)	22 (88.0%)	24 (96.0%)	

## DISCUSSION

In this study, sudden NCPAP weaning was associated with significantly shorter duration of NCPAP use (p=0.002) and a reduced length of hospital stay (8.52 ± 5.65 days) compared with gradual (12.32 ± 5.73 days) and pressure weaning (11.80 ± 5.55 days, p=0.041). None of the infants in the sudden weaning group required reintubation, suggesting that this strategy can provide both clinical and resource benefits. Several other researchers support the advantages of sudden weaning, like Abdel-Hady et al.,^17^ and McMorrow et al.,^18^ noted that sudden weaning shortened CPAP use, hospital stay, and risk of bronchopulmonary dysplasia (BPD), provided infants met strict readiness criteria. Jensen et al.,^11^ in a large multicenter RCT, also found no differences in NCPAP duration, attempts, or hospitalization between sudden and gradual pressure reduction strategies, in contrast to the present results where hospitalization was significantly shorter in the sudden weaning group. These findings are consistent with a study from the USA which showed that sudden weaning led to fewer days on positive pressure support compared with gradual weaning (12 vs. 17 days, p=0.03), with a comparable success rate of 96% versus 88% and 92% for gradual and pressure weaning, respectively (p=0.581).^19^

Broom et al.,^20^ reported that sudden discontinuation reduced overall weaning duration without negatively impacting weight gain, feeding milestones, or caffeine cessation. These observations align with our results, underscoring the importance of using well-defined clinical stability benchmarks before attempting sudden withdrawal. By contrast, studies such as Singh et al.,^21^ Rastogi et al.,^16^ and Soe et al.,^22^ applied less comprehensive or fewer stability parameters, which may explain variable outcomes. Predefined readiness criteria are crucial because premature withdrawal increases the risk of failure, prolongs CPAP use, and adds to the oxygen requirement due to increased respiratory effort.^21^–^23^ Differences across studies may also be influenced by the infant’s characteristics. Infants born before 28 weeks may benefit more from pressure weaning, as suggested by higher first-attempt success in this group.^24^ Clinical conditions such as prior intubation, anaemia, infection, or gastroesophageal reflux can further delay readiness for successful discontinuation.^25^ These factors need to be accounted for when tailoring weaning approaches.

In Pakistan, there is a paucity of data on NCPAP weaning practices, making the present study a valuable addition to local evidence, and it is the need of the hour. It highlights that sudden weaning, when guided by stability criteria, can reduce both NCPAP duration and hospitalization without increasing adverse outcomes. The present findings may help in the development of guidelines about the utilization of various weaning strategies of NCPAP among preterm infants.

### Limitations:

This study was conducted at a single tertiary care center, and most enrolled infants were from an urban population, which may limit the generalizability of the findings to other NICU settings and rural populations. Clinicians were not blinded to allocation, and the randomization process was visible to the researcher. Extremely preterm infants were excluded.

## CONCLUSION

Infants subjected to sudden NCPAP weaning had a significantly shorter overall duration of NCPAP, leading to a significantly reduced total duration of hospitalization compared to those undergoing gradual or pressure weaning. Success rates for sudden, gradual, and pressure NCPAP weaning varied but without any significance.

### Recommendations:

Future studies should include extremely preterm infants and assess long-term outcomes such as BPD, feeding success, and neurodevelopment to establish comprehensive guidelines.

### Author’s Contribution:

**RA:** Data collection and synthesis, drafting, responsible for data’s integrity, critical review,

**HR:** Concept & design, Data collection and analysis, proof reading, critical review.

**TA and SS:** Data collection and synthesis, data analysis, proof reading.

All authors have read and approved the final version of the manuscript.
